# Uncovering the potential virulence factors of emerging pathogens using AI/ML-based tools: a case study in *Emergomyces africanus*

**DOI:** 10.3389/fcimb.2025.1716723

**Published:** 2025-12-02

**Authors:** Peter F. Farag, Karema S. Abdel-monem, Hibah M. Albasri, Areej A. Alhhazmi, Rana H. Ismail

**Affiliations:** 1 Department of Microbiology, Faculty of Science, Ain Shams University, Cairo, Egypt; 2 Department of Microbiology and Biochemistry, Faculty of Science, Benha University, Al-Qalyubia, Egypt; 3 Department of Biology, College of Science, Taibah University, Al-Madinah, Saudi Arabia; 4 Clinical Laboratory Sciences Department, Applied Medical Sciences, Taibah University, Al-Madinah, Saudi Arabia

**Keywords:** AI tools, emerging pathogens, *Emergomyces*, pathogenicity, virulence factors

## Abstract

**Background:**

We are currently in the era of artificial intelligence (AI), which has become deeply embedded across nearly all scientific disciplines. Harnessing this revolutionary technology to predict virulence factors of emerging pathogens can improve our understanding of their pathogenicity, especially since the majority of these pathogens’ proteomes are composed of hypothetical or uncharacterized proteins. Moreover, emerging orphan proteins were expressed from novel open reading frames. Therefore, this study aimed to develop a pipeline for predicting and annotating the species-specific secreted protein structures of these pathogens, with *Emergomyces africanus* selected as a model organism.

**Methods:**

The proteome of *E. africanus* CBS 136260 was retrieved from the NCBI database. The secretome of this fungus was predicted by ML-based SignalP and Phobius tools, targeting signal peptide (SP) bearing proteins. Species-specific proteins were detected using BLASTp (sequence level) and AFDB clusters (structure level). AlphaFold2, an AI-based system, was used to build structural models of hypothetical proteins specific to *Emergomyces*. DeepFRI was used to anticipate functional annotation of these proteins based on their structures, while the DALI server was used to detect homologous similarity. Candidate proteins were applied to molecular docking analysis against MHC-II.

**Results:**

The structure modeling and homologous matching revealed several protein domains similar to toxins (scorpion toxin-like, cytolysin, CARDS toxin, defensin-like), allergens, adhesins, hydrolytic enzymes, and inhibitors. Novel domains with putative functions (ion binding, proteolysis, transferase activity, and protein binding) were also discovered. In immunoinformatics and molecular docking studies, a cytolysin like-containing protein (Gene ID: ACJ72_08076) outperformed the other selected proteins in binding to MHC-II (Docking score = −318.74) with a confidence score = 0.96.

**Conclusion:**

The findings suggest that AI and ML tools can be employed in the preliminary stage to explore host-pathogen interactions and anticipate novel virulence genes.

## 1 Introduction

The emergence of novel pathogens is one of the most pressing issues confronting humanity in recent years, which can be attributed to several factors, including climate change, natural disasters, evolution, and human behavior. This phenomenon poses a serious global health risk and emphasizes the challenge of determining their pathogenicity mechanisms (Seal et al., 2021**;**Asim et al., 2022**;**Casadevall, 2023**;**Seidel et al., 2024). Historically, human fungal infections have been a neglected and underestimated field of disease research compared to bacterial and viral infections (Rodrigues and Nosanchuk, 2020**;**Brown et al., 2024). Among the growing list of fungal pathogens with increasing clinical relevance is *Emergomyces africanus*, an emerging threatening pathogen in Africa (Hoving, 2024).

*E. africanus* is a thermally dimorphic airborne fungus that belongs to the Ajellomycetaceae family. It was first identified in South Africa in 2013, causing emergomycosis (severe systemic disease) among HIV immunocompromised patients (Albergoni et al., 2024). An infection is induced by inhaling conidia from soil, which then modifies into a yeast-like phase in human lungs (Schwartz et al., 2018). The pathogenesis of this infection and the virulence factors secreted by the fungus remain unclear, leading to the complication of disease management (Samaddar and Sharma, 2021**;**Ibe et al., 2023).

Pathogenic fungi, like other microbial pathogens, secrete numerous types of proteins (adhesion molecules, hydrolytic enzymes, toxins, etc.), which represent the virulence factors of pathogens that enable them to interact and infect the host (Ranganathan and Garg, 2009**;**Bouqellah et al., 2023). Roughly 20–60% of these secreted proteins (secretome) are classified as hypothetical proteins, which are expected to play critical roles in host-pathogen interactions (HPIs) but do not match known experimental proteins (Ashrafi et al., 2019**;**Farag et al., 2025). Another important category of proteins is called an orphan, which has no close homology to any other proteins and is specific to a single species or lineage (Liu et al., 2023).

All of the foregoing concerns compel us to focus our attention on the applications of AI and machine learning (ML), a subfield of AI, in biological sciences for attempting to solve these obstacles (Bhardwaj et al., 2022). Identifying fungal protein-protein interactions and predicting the 3d protein structures could help us understand pathogenesis and develop treatments for infectious diseases (Humphreys et al., 2024). To address the limitation of sequence annotations for the aforementioned proteins, generative AI-based tools, such as AlphaFold2, were utilized for protein structure prediction (Jumper et al., 2021).

John Jumper and Demis Hassabis were awarded a Nobel Prize in chemistry (2024) for developing AI-based AlphaFold (Callaway, 2024). This tool facilitated the annotation of proteins by generating reliable 3D structure models for sequences of up to 1,400 amino acids and assigning average confidence scores (Predicted Local Distance Difference Test, pLDDT 0-100) per protein (Pereira et al., 2021). The AlphaFold2 Colab (online version) is a simplified version of AlphaFold2 that enables protein structure and complex prediction using AlphaFold2 and AlphaFold2-multimer with the same evaluation criteria as AF2. Sequence alignments/templates are generated through MMseqs2 and HHsearch (Mirdita et al., 2022).

This work aims to use AI and ML tools to investigate the virulent proteins secreted by *E. africanus*, a representative emerging pathogen, serving as a roadmap for studying other pathogens. AI provides a powerful alternative for predicting structural traits and guiding initial investigations into these proteins, filling the gap due to the paucity of experimental data on these fungal groups.

## 2 Materials and methods

**Figure 1:**
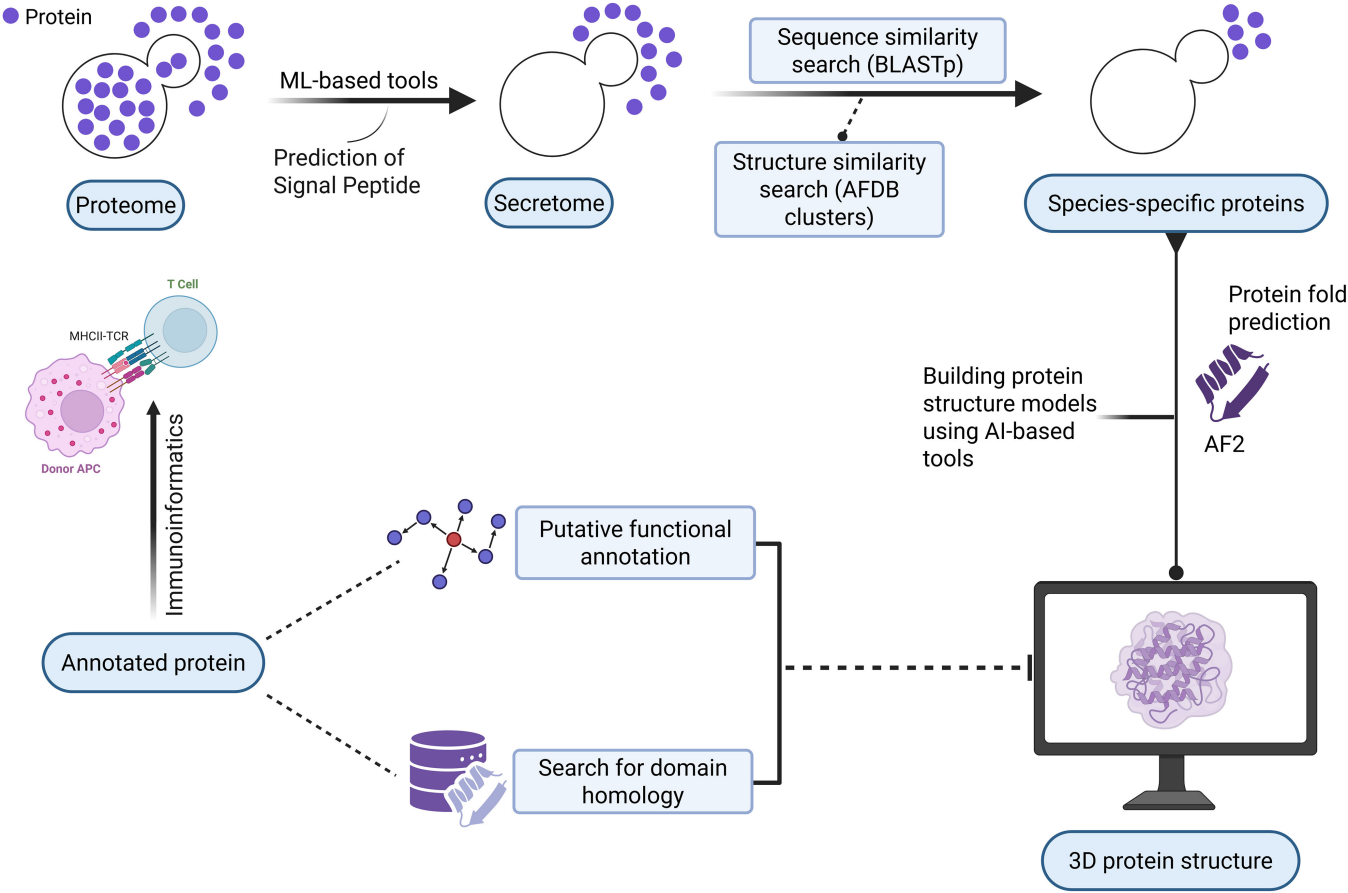
Schematic diagram depicts the workflow of this study. Created with BioRender.com.

**Figure 1** illustrates the schematic workflow of this study. This approach can be used as a preliminary screening to detect potential virulence factors in new pathogens. We applied the following detailed steps to a model of the emerging pathogen *E. africanus* to assess this pipeline.

### 2.1 Retrieval of *E. africanus* proteome and secretome prediction

The only proteome (the total proteins produced by the organism) data of *Emergomyces africanus* (strain CBS 136260) were submitted to the NCBI database (Genbank accession no. GCA_001660665.1) in June 2016 (https://www.ncbi.nlm.nih.gov/datasets/gene/GCA_001660665.1/, accessed on 14 January 2025) (Dukik et al., 2017). The genome of this strain has a length of 29.7 Mb and contains 8,860 genes, which encode 8,769 proteins. Out of these, 7,699 (87.7%) were classified as hypothetical proteins, whereas 1,070 (12.3%) were fully annotated proteins. The secretome was then predicted by looking for proteins that carry a signal peptide (SP) lacking a transmembrane helix using SignalP v6.0 and the Phobius web servers (Käll et al., 2007**;**Teufel et al., 2022).

### 2.2 Screening of species-specific proteins

From the secretome, *Emergomyces*-specific proteins (including *E. africanus* proteins and Genus-specific proteins) were identified by comparing their sequence and structure similarities to those of other microbial proteins. Proteins were clustered according to structural similarity using AFDB/MMseqs2 via AFDB clusters (Steinegger and Söding, 2018**;**Barrio-Hernandez et al., 2023), and sequence similarity using the NCBI BLASTp 2.16 (Database is *nr* and *E* value < 1 x 10^−20^) via SkyBLAST (https://sky-blast.com/blast/p).

### 2.3 Prediction of domains and homologous similarity searching

The domains of selected protein sequences were predicted using InterPro 105 (Blum et al., 2025). AF2/ColabFold v1.5.5 was applied for designing 3D structures of protein domains after removing the SP sequence (Mirdita et al., 2022**;**Liu et al., 2023). pLDDT confidence scores below 70 are not acceptable, while scores above 90 are considered precise enough to be comparable to crystallography protein data. Proteins’ homologous structural similarity can be assessed using predicted template modeling (pTM) scores that range from 0 to 1 (Tunyasuvunakool et al., 2021**;**Varadi et al., 2022). ChimeraX v1.6.1 was used to visualize the best 3D protein structure model created by AF2, which had the highest pLDDT value (Meng et al., 2023). For homologous structural searches, the RUPEE web server was utilized to match similarity against SCOPe v2.08 (Top aligned, Full length). Two structures were deemed evolutionarily related if their TM scores surpassed 0.5 (Ayoub and Lee, 2019, 2021). The DALI service (Holm et al., 2023) was applied for all-against-all comparisons of more than two proteins and pairwise structural similarity against the PDB database (DALI Z-score similarity cutoff = 2). The structure alignment was confirmed through the output TM-score built by the US-align server (Zhang et al., 2022).

### 2.4 Protein function anticipation and physicochemical analysis

The DeepFRI (score > 0.5), a graph convolutional network tool, was used to annotate protein functions based on their predicted structures. The output data includes Gene Ontology (GO) terms for molecular function (MF) and biological process (BP) (Gligorijević et al., 2021). Protein sequences were retrieved in FASTA format from the NCBI database, and enriched GO terms were predicted using the Argot^2.5^ web service (score > 100) (Lavezzo et al., 2016). The DL-based ToxinPred 3.0, ToxiPep, and NTxPred2.0 web servers were utilized to evaluate the potential toxicity of knottin fold-containing proteins (Rathore et al., 2024**;**Guan et al., 2025**;**Rathore et al., 2025**),** with NTxPred2.0 specifically assessing neurotoxicity. The physicochemical parameters were characterized by the Multiple Protein Profiler (MPP v.1) (Sganzerla Martinez et al., 2024).

### 2.5 Immunoinformatics and molecular docking analysis

The candidate proteins were tested for their ability to activate helper T-cells and trigger the release of interleukins using ML-based web servers. The IFNepitope2 (threshold 0.5), IL-6Pred (threshold 0.1), and IL17eScan (threshold 0.2) can predict IFN-γ, IL6, and IL17-inducible peptides, respectively (Gupta et al., 2017**;**Dhall et al., 2023**;**Dhall et al., 2024). The Major Histocompatibility Complex II (MHC-II) allele was predicted using the EpiDOCK v1.1 web server (Atanasova et al., 2013). Protein-protein docking was executed between the receptor MHC-II (PDB: 4OV5) and target proteins using the HDOCK server (Yan et al., 2020). The top 10 models, generated from HDOCK, are ranked according to the docking scores, and the highest-ranked model for each docking prediction was retrieved and evaluated with the PRODIGY web server (https://rascar.science.uu.nl/prodigy/), which assesses the binding affinity (Xue et al., 2016). The number and type of contacts were predicted using the COCOMAPS 2.0 web server (https://aocdweb.com/BioTools/cocomaps2/) (Vangone et al., 2011**;**Romina, 2025).

## 3 Results and discussion

The emergence of novel pathogens and drug resistance represents a risk to human health, necessitating innovative approaches to investigate these outbreaks. With significant mortality rates (40-50%) and a paucity of available data (Vinayagamoorthy et al., 2023), this study sheds light on the virulence genes of *Emergomyces africanus*. To date, AI and ML are arguably the most promising tools in tackling several biological issues, including the study of host-pathogen interactions (Fisch et al., 2019**;**Rawas, 2024). Despite the crucial importance of experimental studies, these computational tools can dramatically minimize the number of trials required for HPI identification by predicting potential pathogen virulence factors (Yakimovich, 2021). For this reason, the model performance of several structural modeling tools (AF2, trRosetta, and Multifold) was tested for random sequences at the beginning of work using LDDT confidence scores as an evaluation criterion. The findings revealed the exceptional performance of AF2 over other tools. The methods section outlines the steps taken to do that, and the results are now displayed as follows:

### 3.1 Prediction of secreted proteins

A Genome-based Secretome comprises proteins that are defined by the presence of a short N-terminal amino acid sequence known as a signal peptide (Yang et al., 2025). These proteins target and damage hosts by disrupting cellular processes and inhibiting their protein synthesis (Fontana et al., 2011). The advent of various ML-based methods has enabled the prediction of SP-bearing proteins throughout the proteome of an organism (Käll et al., 2007**;**Teufel et al., 2022). SignalP 6.0 and Phobius web servers were used to infer the secretome of *E. africanus*. From a total of 8,769 proteins (proteome), only 394 (4.5%) make up the secretome of *E. africanus* (**Supplementary Table S1**). According to several studies, the fungal secretome accounts for 5–10% (less than 1000 proteins) of the overall fungal proteome (Varona et al., 2020**;**Jia et al., 2023).

### 3.2 Characterization of proteins related to *E. africanus*

To detect and identify secreted proteins specific to *E. africanus*, we examined the homology of all secreted proteins using BLASTp and AFDB clusters. The result of this investigation yielded 62 hypothetical orphan proteins. These proteins were divided into three categories:

#### 3.2.1 Annotation *E. africanus*-specific proteins

**Figure 2:**
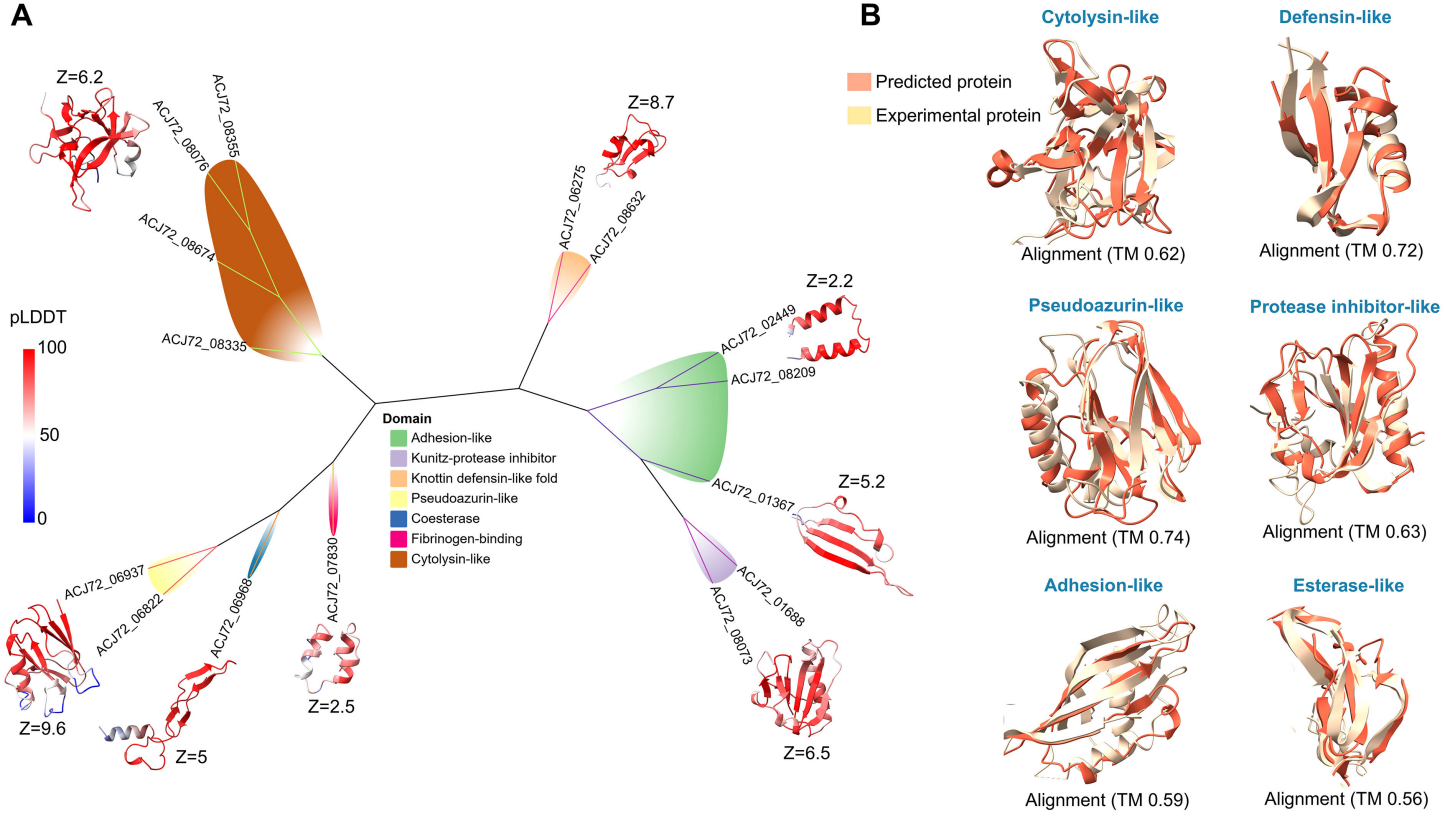
Structure-based clustering of *E. africanus* specific proteins. **(A)** Unrooted similarity dendrogram of 15 proteins with their evolutionary Z-score values. **(B)** The alignment scores between them and their homology known experimental proteins.

Based on sequence and structural homology, twenty-five proteins are secreted exclusively by *E. africanus*. InterPro failed to annotate most of these proteins. At the same time, AF2 successfully designed 3D structures for 15 protein domains with high confidence scores (pLDDT > 70) (**Figure 2**). With structure-based similarity, different protein clusters and singletons share folds similar to known virulence factors (**Figure 2A**). A group of genes (ACJ72_08355, ACJ72_083076, ACJ72_08674, and ACJ72_08335) encode protein domains similar to bacterial cytolysin (PDB: 4OWJ) with an average aligned TM score equal to 0.62 (**Figures 2A, B**). Cytolysin is a pore-forming toxin, mainly secreted by pathogenic bacteria as a virulence factor to destroy cells and disseminate within the human body (Kaus et al., 2014**;**Krone et al., 2024). After matching the SCOPe fold database v2.08, we investigated another set of proteins (Gene IDs: ACJ72_06275 and ACJ72_08632) that resemble a knottin toxin-like defensin (SCOPe: d1n4na), with a TM superposition between them equal to 0.72 (**Figure 2B**). The SCOPe database classifies structural cysteine-rich knotted proteins (30–50 amino acids) linked by disulfide bridges as inhibitor cystine knots (ICKs) or knottins. Toxins, lectins, and inhibitors from living organisms are members of these families (Postic et al., 2018**;**Chandonia et al., 2022**;**Li et al., 2024). The toxicity prediction was implemented for the two knottin folds using ToxinPred 3.0, ToxiPep, and NTxPred2.0 web servers. The results demonstrated the high potential neurotoxicity of these proteins (scoring 0.8, 0.99, and 0.94, respectively). Two proteins (Gene IDs: ACJ72_08073 and ACJ72_01688) showed evolutionary relations to kunitz-type protease inhibitors (**Figures 2A, B**). This protein family inhibits serine proteases, which may play a role in infection (de Magalhães et al., 2018). Three proteins (ACJ72_02449, ACJ72_08209, and ACJ72_01367) have putative adhesion functions that bind to host cells and initiate the infection process (Bisht and Meena, 2019). AFDB and DALI assume a singleton protein (Gene ID: ACJ72_06968) has a cholinesterase-like domain (PDB: 5FPQ), and InterPro predicts an alpha/beta hydrolase fold (IPR029058) for this domain with E-value = 5.4 X 10^-7^. Cholinesterases, which belong to the alpha/beta hydrolase superfamily, are important for nerve impulse termination by hydrolyzing choline esters (De Boer et al., 2021). It was discovered that a new cholinesterase-like adhesion family has a role in cell-cell interactions, independent of the catalytic domain of cholinesterases (Krejci et al., 1991**;**Gilbert and Auld, 2005). It is astonishing the high degree of evolutionary similarity (Z = 9.6) between proteins (Gene IDs: ACJ72_06937 and ACJ72_06822) and pseudoazurin (PDB: 5Y23). Pseudoazurin (cupredoxin) is an electron donor to copper-containing nitrite reductases during the denitrification pathway (Ferroni et al., 2014). Denitrifying bacteria are the main producers of this protein, which may have an indirect role in host relationships (Laming et al., 2012). Horizontal gene transfer may be the primary reason for the emergence and prevalence of pathogens through facilitating the transfer of genetic material, including virulence genes, between different organisms (de la Casa-Esperón, 2012**;**Juhas, 2015).

#### 3.2.2 Annotation of *Emergomyces*-specific proteins

**Figure 3:**
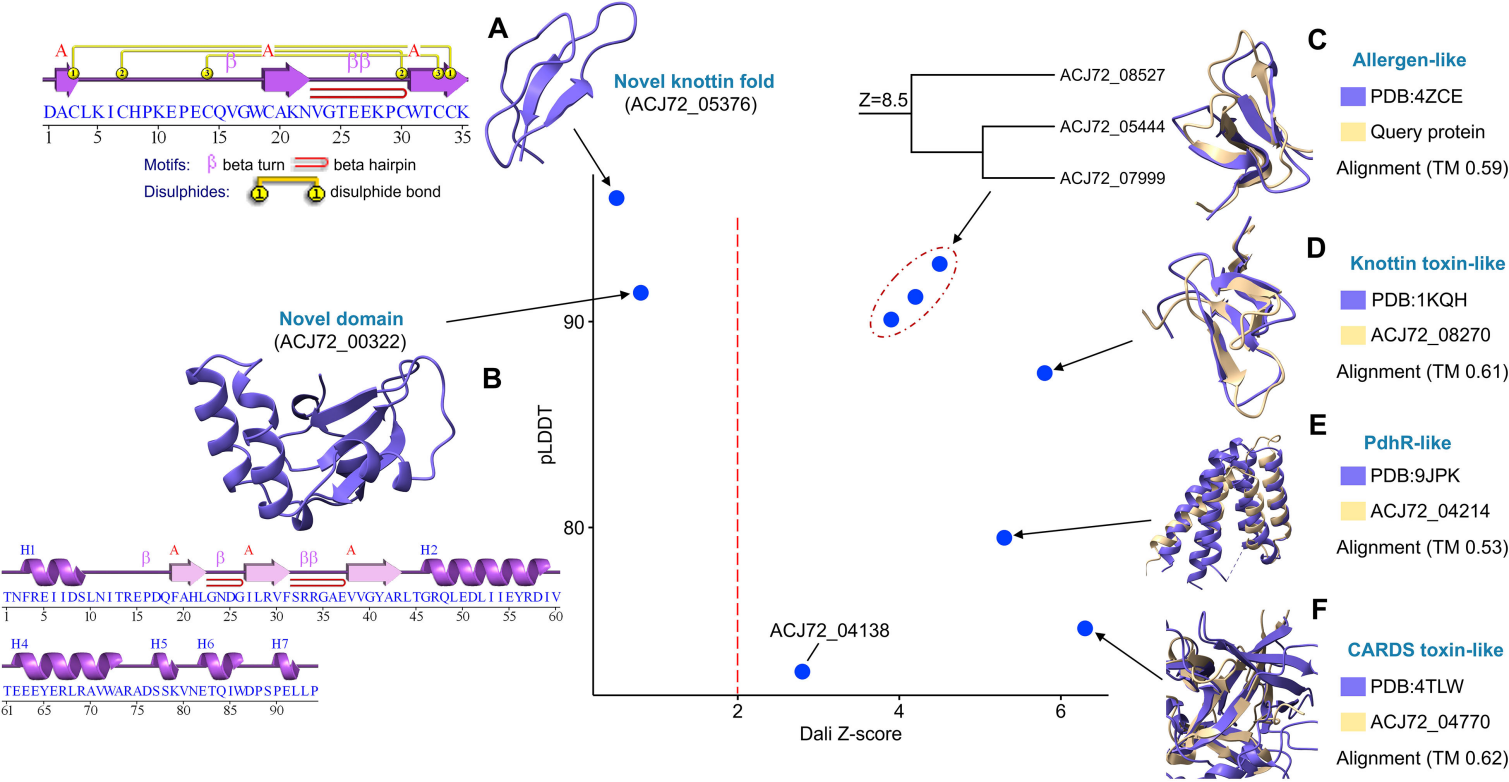
Scatter plot illustrates structure-based homology between *Emergomyces*-specific proteins and known experimental proteins, where the evolutionary relationships between them were measured by Dali Z-score (Cutoff=2) and the quality of proteins was calculated by pLDDT>70. The different protein families were mentioned in the figure from **(A–F)**.

Sixteen proteins were detected to be associated with different species of *Emergomyces*, mainly *E. africanus* and *E. pasteurianus*. Nine proteins were structurally constructed with different confidence and Z scores (**Figure 3**). We discovered a novel protein (Gene: ACJ72_05376, 62 a.a.) that contains a knottin motif sequence (35 a.a.) rich in cysteines (7C) connected with 3 disulfide bonds, and forms one beta hairpin (**Figure 3A**). Despite the very high confidence score (pLDDT=96), it lacks a predicted homology to known proteins (Z<2). The putative structure-based biological process functional annotation was described as “interspecies interaction between organisms” (GO:0044419), which suggests that this protein permits the interaction between the pathogen and host. ToxinPred 3.0 and ToxiPep DL-based tools were used to predict the potential toxicity of this motif, whereas NTxPred2.0 was utilized to evaluate the possibility of its neurotoxicity. ToxiPep predicts that it is a toxin with a high probability value (99.79%), and a maximum score (1.0) was also reported by ToxinPred. This motif may have putative neurotoxin activity (Score=0.7) according to the NTxPred2.0 web server. We captured another domain (Gene: ACJ72_00322), which has no homology to any experimental protein (**Figure 3B**). It is presumed to be a “nucleic acid binding” molecular function (GO:0003676). During the infection, some pathogens can produce proteins that interact with host nucleic acids (DNA or RNA) to facilitate their pathogenicity (Pagliuso et al., 2019). Three proteins (**Figure 3C**) have a resemblance to dust mite allergens (PDB: 4ZCE), which may cause allergic respiratory diseases like asthma and trigger inflammatory responses (Keshavarz et al., 2021). Two toxin-like proteins were found with putative homology with high pLDDT values (**Figures 3D, F**). The first protein is similar to atracotoxin (ACTX-HI neurotoxin) produced by the Australian funnel-web spider *Hadronyche infensa*, targeting the voltage-gated sodium channels with insecticidal and mammalian toxicity (Rosengren et al., 2002). The second one resembles Community-Acquired Respiratory Distress Syndrome Toxin (CARDS TX) that was secreted by *Mycoplasma pneumoniae*, causing tracheobronchitis (Becker et al., 2015). A protein encoded by the ACJ72_04214 gene is structurally like pyruvate dehydrogenase regulator (PdhR) (**Figure 3E**), which represses the transcription of certain genes (Ogasawara et al., 2007). This protein may play a potential role in different diseases by affecting the pyruvate metabolic pathway (Ma et al., 2021).

#### 3.2.3 Annotation of structure-based *E. africanus*-specific proteins

**Figure 4:**
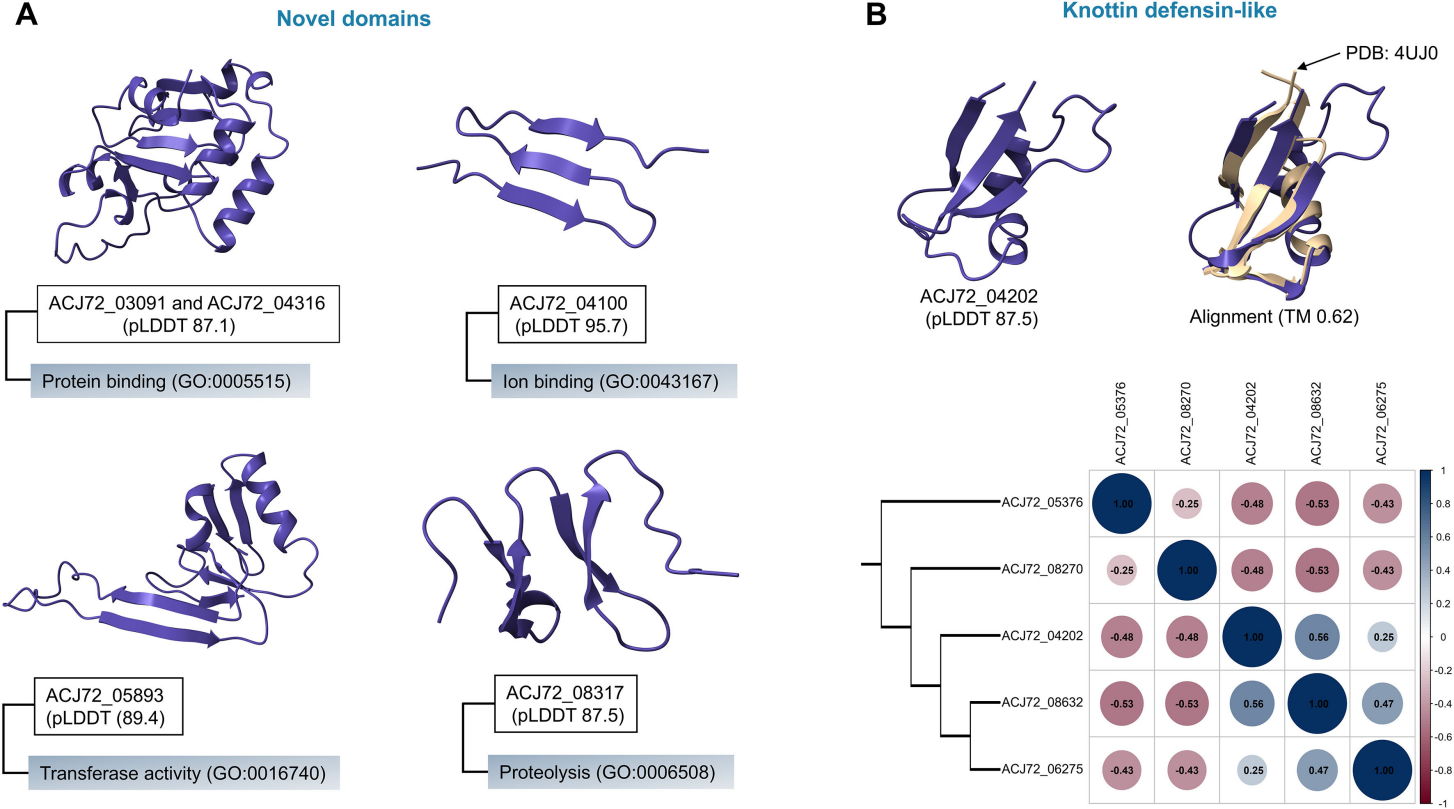
Shows the predicted novel domains and motifs for several proteins. **(A)**. The defensin-like domain containing protein (ACJ72_04202) and its relation to other proteins contain knottin fold through dendrogram and the similarity matrix **(B)**.

The remaining proteins are structurally linked to *E. africans* but exhibit sequence similarities to members of the Ajellomycetaceae family (*Histoplasma*, *Blastomyces*, *Paracoccidioides*, etc.) with an E-value close to the BLASTp threshold value. Several new domains and motifs were observed with putative functional annotation (**Figure 4A**). Two proteins (Gene ID: ACJ72_03091 and ACJ72_04316) were produced by *E. africanus* (BLASTp E-value = 10^−121^) and *Histoplasma capsulatum* (BLASTp E-value = 10^−25^). These proteins share the same new fold that is expected to have a protein-binding function (GO:0005515). A new motif-containing protein (Gene ID: ACJ72_04100) was predicted to have a high confidence score (pLDDT = 95.7) and a putative ion-binding function (GO:0043167). Several pathogens from the same family can secrete a novel protein (Gene ID: ACJ72_05893) with a domain that has potential transferase activity (GO: 0016740). Moreover, a novel detected domain in a protein encoded by a Gene ACJ72_08317 has proteolytic activity (GO: 0006508). The previously predicted functions typically contribute to pathogenicity and can be categorized as virulence factors (Urban, 2009**;**Bagchi, 2015**;**Calmes et al., 2015). **Figure 4B** describes a floral defensin-like protein, a plant defensin that attacks its adversaries’ cell membranes (Baxter et al., 2015). This figure also explains the distribution of all knottin-folded motifs revealed in this study, as well as the unique fold motif (Gene ID: ACJ72_05376).

### 3.3 Physicochemical properties of *Emergomyce*s-specific proteins

**Figure 5:**
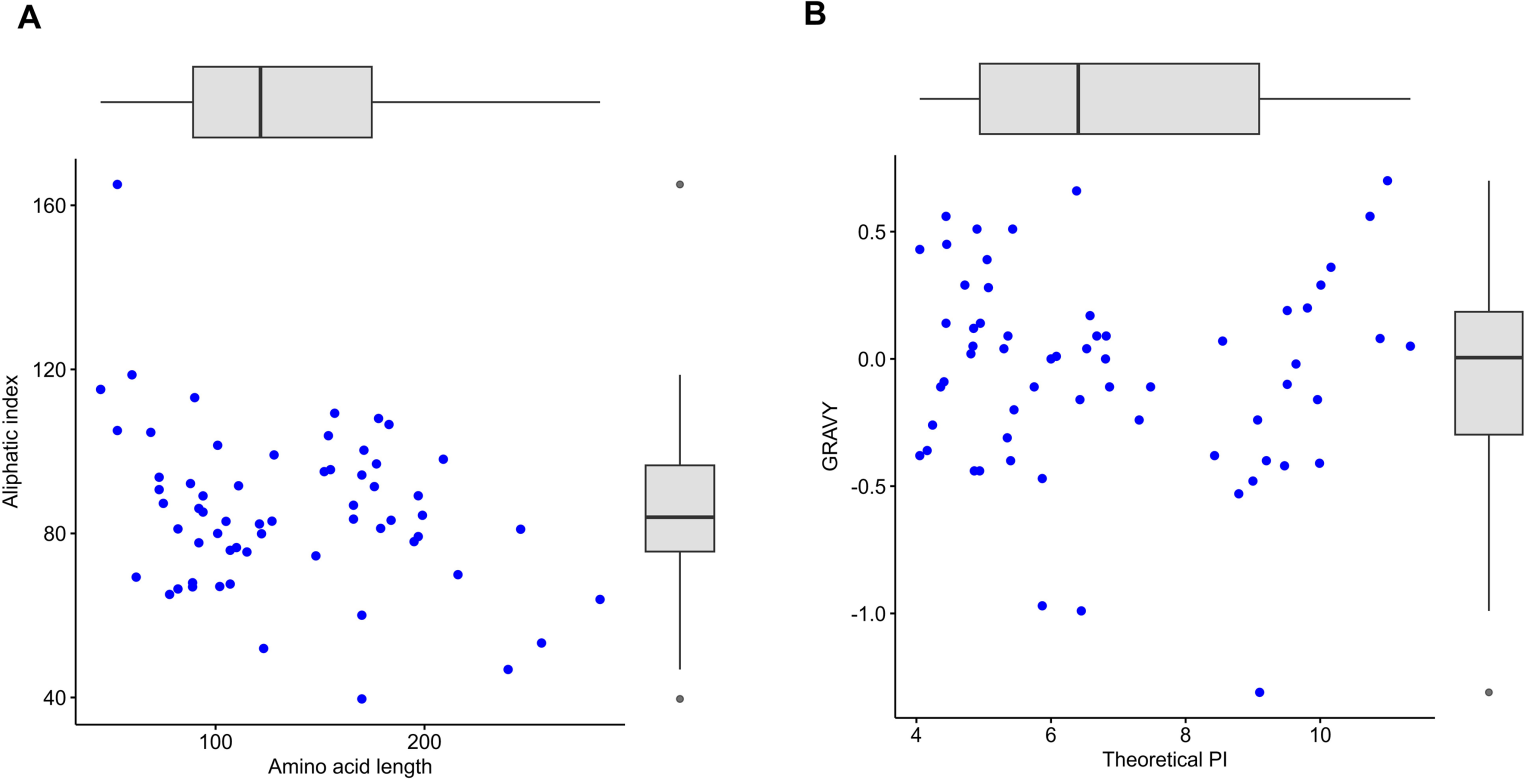
Physicochemical characteristics of the target 62 secreted proteins. **(A)** protein length (a.a.) vs. Aliphatic index (AI). **(B)** Theoretical pI vs. the GRAVY values.

The physical and chemical properties of the sixty-two proteins were analyzed using MPP v.1, which can determine their behavior and stability under various conditions (Quintero-Quiroz et al., 2022). The output data included the molecular weight (MW), theoretical isoelectric point (pI), amino acid length, charge at pH7, aliphatic index (AI), grand average hydropathicity (GRAVY), and aromaticity (**Supplementary Table 2**). The results revealed that these proteins are small-secreted proteins since their lengths ranged from 45 to 284 amino acids (**Figure 5A**). Small Secreted Proteins (SSPs) are less than 300 amino acids, with the majority lacking known domains and playing a vital role in pathogenicity (Kim et al., 2016**;**Feldman et al., 2020). The molecular weight (MW) ranged from 4.84 kDa to 30.91 kDa (**Supplementary Table S2**). The Aliphatic index was used to measure the thermostability of proteins; a higher aliphatic index value implies that the protein is more stable at high temperatures (Kaur et al., 2020). The aliphatic index values of the examined proteins revealed the great thermostability of most proteins (50-165) throughout vast temperature ranges, with an average of 90 (**Figure 5A**). For the theoretical isoelectric point (pI) values, the majority of proteins (60%) tended to be acidic, whereas 20 proteins (ranging from 8.2 to 11.3) were basic (**Figure 5B**). The pI of any protein is the pH at which the carried net charge equals zero. It establishes a protein’s activity, stability, and solubility as well as how it interacts with other molecules in various pH environments. As a result, pI values have long been employed to identify distinct proteins in processes such as protein isolation, separation, purification, and crystallization (Enany, 2014**;**Tokmakov et al., 2021). According to the GRAVY analysis, most proteins showed negative scores (39 proteins), while 23 proteins showed positive scores (**Figure 5B**). The positive values of the GRAVY indicated the hydrophobicity of the proteins (membrane type), while the negative scores indicated hydrophilicity (globular type) (Magdeldin et al., 2012).

### 3.4 Immunoinformatics and molecular docking analysis

**Figure 6:**
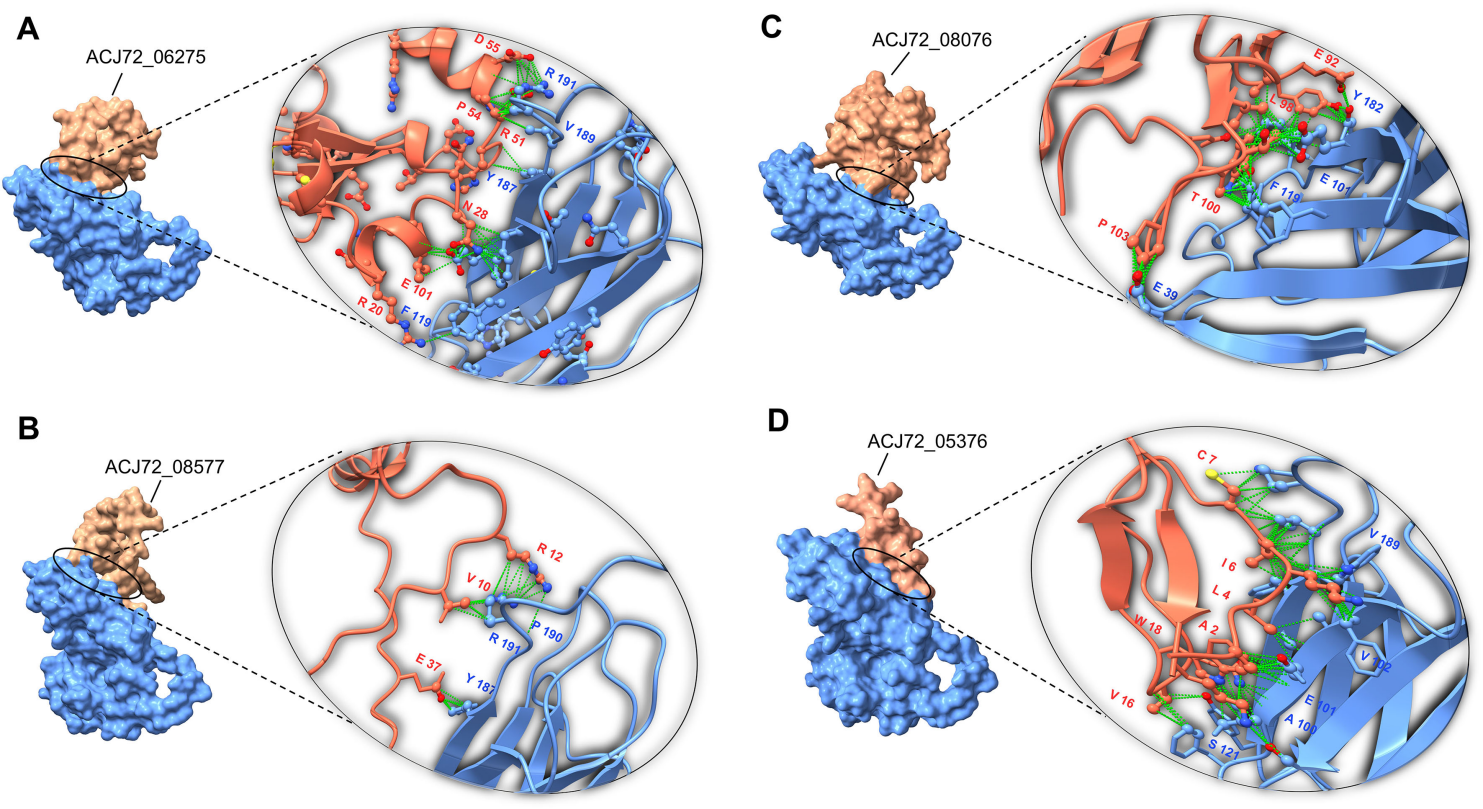
Protein-protein molecular docking between MHC-II (PDB: 4OV5) and **(A)** ACJ72_06275. **(B)** ACJ72_08577. **(C)** ACJ72_08076. **(D)** ACJ72_05376.

**Figure 7:**
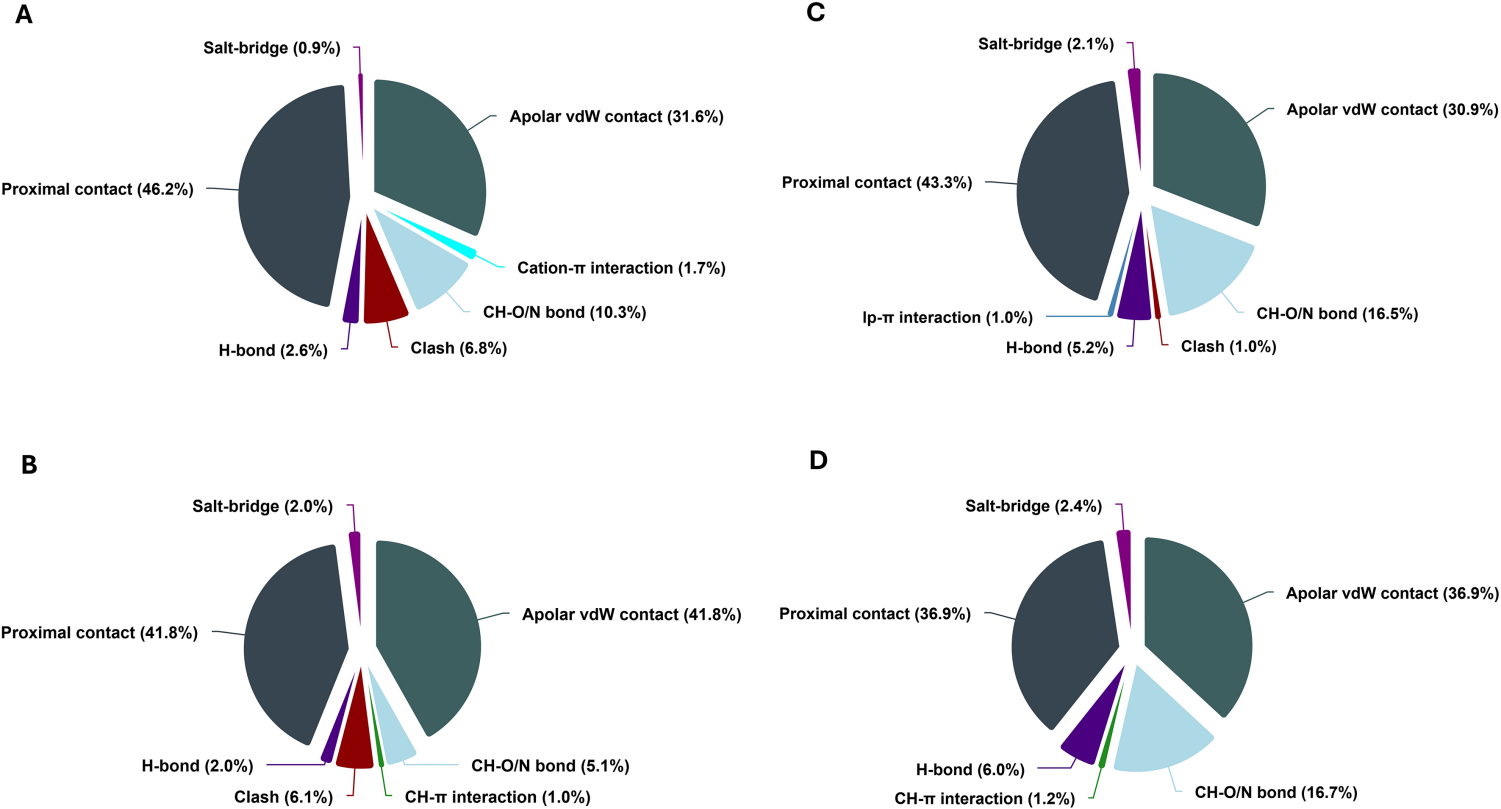
Pie charts showing the atomic interactions distribution between MHC-II (PDB: 4OV5) and **(A)** ACJ72_06275. **(B)** ACJ72_08577. **(C)** ACJ72_08076. **(D)** ACJ72_05376.

Immunoinformatics, a critical branch of bioinformatics, provides new perspectives on biological complexity. Their computational algorithms were utilized to anticipate immunogenic epitopes that are recognized by B and T lymphocytes via MHC class I and II molecules (Raoufi et al., 2020). Höft et al. (2024) studied the murine immune response against *E. africanus* (CBS 136260). According to their investigations, pro-inflammatory cytokines such as IFN-γ, IL-6, and IL-17 were produced at higher levels. Furthermore, their findings support a T-helper type-1 (Th1) immunological response to *E. africanus*. Here, In-silico immunoanalysis was performed on *Emergomyces*-specific proteins to identify those that bind to the MHC-II receptor and induce the production of IFN-γ, IL-6, and IL-17. Various tools facilitate primary screening of cytokine-inducing epitopes in the candidate proteins (See “Methods section”). The most potent proteins that can induce the release of IFN-γ, IL-6, and IL-17 are shown in **Table 1**. The Th1 response is mostly linked to IFN-γ (interferon-gamma), which promotes inflammation and cell-mediated immunity. A pleiotropic cytokine, interleukin-6 (IL-6), is implicated in inflammation, hematopoiesis, and a variety of immunological responses. Interleukin-17 (IL-17) is a crucial cytokine that promotes inflammation, especially in response to bacterial and fungal infections. The development of disease and the immune response can be significantly impacted by the interaction of these three cytokines (Teunissen et al., 1998**;**Arican et al., 2005**;**Tanaka et al., 2014). To assess the binding affinity of the best cytokine-inducing proteins to MHC-II, Epidock v1.1 was used to screen the most proper allele (**Supplementary Table S3**). The peptide fragment binds to MHC-II, forming an MHC II-peptide complex, which is essential for activating helper T cells to release cytokines (Purcell et al., 2019). The HDOCK web server was used for assessing the protein-protein complex between MHC-II and the candidate proteins. HDOCK is a web server of a hybrid docking algorithm that combines template-based modeling with ab initio template-free docking. The docking program first samples the putative binding modes between two proteins through a fast Fourier transform (FFT)–based global search method with an intrinsic scoring function. It next uses an enhanced iterative knowledge-based scoring function for protein–protein interactions to assess the sampled binding modes (Yan et al., 2017**;**Yan et al., 2020). **Figure 6** depicts the interaction of MHC-II DR (PDB: 4OV5) with the most effective proteins. The results revealed that most of the proteins share the same active site. The HDOCK docking score between MHC-II and ACJ72_06275 (A) was −273.31 (confidence score=0.92), ACJ72_08577 (B) was −246.73 (confidence score=0.87), ACJ72_08076 (C) was −318.74 (confidence score=0.96), and ACJ72_05376 (D) was −250.72 (confidence score=0.85). The two protein molecules would most likely bind if the docking score was less than −200 and the confidence score was more than 0.7 (Yan et al., 2020). The free binding energy (ΔG) was calculated for the retrieved docked complex using the PRODIGY web server. The binding affinity (ΔG) between MHC-II and ACJ72_06275 (A) was −9.7 Kcal/mol, ACJ72_08577 (B) was −7.9 Kcal/mol, ACJ72_08076 (C) was −11.8 Kcal/mol, and ACJ72_05376 (D) was −9.5 Kcal/mol. In addition, a quantitative mapping of different interaction types, such as hydrogen bonds and van der Waals contacts, was analyzed by the COCOMAPS 2.0 web server. **Figure 7** shows the percentage distribution of atomic interactions for the docked complex, while the detailed residual interface and the types of interactions were illustrated in **Supplementary Table S4**. Furthermore, COCOMAPS 2.0 calculated the buried surface area (BSA) for the analyzed complexes, which is a valuable quantitative indicator of interface stability and complementarity, with a greater BSA indicating a more stable complex (Chakravarty et al., 2013**;**Belapure et al., 2023). The BSA (Å²) of docked complex (A) was 2120, (B) was 1810.2, (C) was 2275.8, and (D) was 1950.6. Finally, it is noticeable that three of the tested proteins are toxin-like, while the fourth (Gene ID: ACJ72_08577) is a small protein (53 a.a.) specific to *E. africanus* containing indistinguishable domain structure without a putative function.

**Table 1:** Peptides screened for the induction of IFN-γ, IL-6, and IL-17.

Gene id	Peptides	IFN-γ	IL-6	IL-17
ACJ72_08577	LGLGLGLELELEQEQ	0.83	0.12	0.7
ACJ72_08076	MWCSSPWASLLLCLV	0.72	0.28	0.36
ACJ72_06275	LRKGKTWDCVNMIGS	0.75	Non-inducer	0.34
ACJ72_05376	ECQVGWCAKNVGTEE	0.74	0.16	0.32

Collectively, various putative toxins were secreted from *E. africanus*. These toxins target different types of cells, including neurons, blood, and lung tissues. This startling finding is consistent with the fact that emergomycosis is a systemic disease involving skin, lungs, liver, nervous system, lymph nodes, and brain (Govender and Grayson, 2019). These proteins were annotated using structure-based AI modeling, which remains elusive for classic sequence-based methods (Al-Fatlawi et al., 2023). Several recent works utilized structural AI modelling tools for identifying and discovering novel protein families (Seong and Krasileva, 2021, 2023**;**Barrio-Hernandez et al., 2023**;**Durairaj et al., 2023). While computational protein structure prediction helps in elucidating the potential virulence genes of *E. africanus*, some limits in our study must be acknowledged. First, AF2 was unable to predict about 35% of protein structures. Second, the structural annotation is insufficient to predict protein functions. Despite these limitations, *in silico* analyses are considered the first step toward future experimental studies.

## 4 Conclusions

We can conclude from this work that AI/ML tools enable the prediction of novel domain folds with putative functions and other potential virulent proteins, shedding light on the significance of studying the diversity of small secreted protein structures among microbial pathogens. These techniques helped to filter and reduce the data, paving the way for further experimental validations. Furthermore, this study can serve as a guide for investigating the pathogenicity of emerging or other pathogens.

## Data Availability

The datasets generated and/or analysed during the current study are available in the NCBI database under the Bioproject accession number PRJNA284519.
